# Dysregulation of valvular interstitial cell let-7c, miR-17, miR-20a, and miR-30d in naturally occurring canine myxomatous mitral valve disease

**DOI:** 10.1371/journal.pone.0188617

**Published:** 2018-01-09

**Authors:** Vicky K. Yang, Albert K. Tai, Terry P. Huh, Dawn M. Meola, Christine M. Juhr, Nicholas A. Robinson, Andrew M. Hoffman

**Affiliations:** 1 Department of Clinical Sciences, Tufts University Cummings School of Veterinary Medicine, North Grafton, Massachusetts, United States of America; 2 Department of Integrative Physiology and Pathobiology, Tufts University School of Medicine, Boston, Massachusetts, United States of America; 3 Department of Biomedical Sciences, Tufts University Cummings School of Veterinary Medicine, North Grafton, Massachusetts, United States of America; Cincinnati Children’s Hospital Medical Center, UNITED STATES

## Abstract

Canine myxomatous mitral valve disease (MMVD) resembles the early stages of myxomatous pathology seen in human non-syndromic mitral valve prolapse, a common valvular heart disease in the adult human population. Canine MMVD is seen in older subjects, suggesting age-related epigenetic dysregulation leading to derangements in valvular cell populations and matrix synthesis or degradation. We hypothesized that valvular interstitial cells (VICs) undergo disease-relevant changes in miRNA expression. In primary VIC lines from diseased and control valves, miRNA expression was profiled using RT-qPCR and next generation sequencing. VICs from diseased valves showed phenotypic changes consistent with myofibroblastic differentiation (vimentin^low+^, α-SMA^high+^), increases in senescence markers (p21, SA-β-gαl), and decreased cell viability and proliferation potential. RT-qPCR and miRNA sequencing analyses both showed significant (p<0.05) downregulation of let-7c, miR-17, miR-20a, and miR-30d in VICs from diseased valves compared to controls. Decreased let-7c, miR-17, and miR-20a may contribute to myofibroblastic differentiation in addition to cell senescence, and decreased miR-30d may disinhibit cell apoptosis. These data support the hypothesis that epigenetic dysregulation plays an important role in age-related canine MMVD.

## Introduction

Non-syndromic mitral valve prolapse (MVP) is a common valvular heart disease with a prevalence of 3% in the human adult population [1]. Although most MVP events are benign, ruptured chordae or poor coaptation of the valve leaflets can lead to mitral regurgitation (MR) with ensuing congestive heart failure (CHF). Currently, medical management of MR is of limited value, and mitral valve repair or replacement are most effective when provided before the onset of CHF [1].

The approach to exploring molecular targets for MVP would be greatly aided by a realistic animal model. Naturally occurring myxomatous mitral valve disease (MMVD) in dogs closely resembles early stages of non-syndromic MVP in humans structurally, and functional consequences are similar, i.e., the development of cardiac enlargement and remodeling, and CHF at the end-stage of the disease. MMVD is the most common acquired cardiac disease and the most common cause of CHF in dogs, comprising 2/3 of all canine cardiac cases [2]. This disease is clearly age-related, with a lifetime prevalence in older dogs nearing 100% [2]. MMVD leads to MR, cardiac enlargement, and contractile dysfunction in the later stages. Once in CHF, dogs are reported to have a median survival time between 1 and 9 months [2].

The major cellular-molecular pathology in MMVD involves resident valve interstitial cells (VICs) and their dysregulated matrix synthetic products. Dysfunction in valvular ECM regulation, inflammatory responses, epithelial-to-mesenchymal transition, and age-related cell senescence have been implicated in MMVD [3, 4]. Both serotonin and TGF-β stimulation have been implication in disease development as well [5, 6]. In diseased valves, overproduction of glycosaminoglycan and proteoglycans are observed, leading to disruption and fragmentation of the native collagen and elastin fibers within the valves [7, 8], which is referred to as myxomatous changes [9]. It is the phenotypic transition of VIC from fibroblastic-like cells (vimentin^high+^, α-SMA^low+^) to myofibroblastic-like cells (vimentin^low+^, α-SMA^high+^) [10] that appears to be associated with myxomatous pathology. The exact mechanism that leads to myofibroblastic transformation is unknown. We therefore investigated the involvement of epigenetic dysregulation, namely the effects of changes in miRNA expression, may have in precipitating this process.

The role of non-coding RNA, such as miRNA, which are key players in epigenetic regulation [11], have been explored in canine heart diseases; however, studies in canine MMVD have been limited to studies of circulating biomarkers. For example, we showed that changes in plasma exosomal miRNA (miR-9, miR-181c, miR-495, miR-599) that are known to regulate fibrosis, cardiomyocyte energetics, and mitochondrial function are detected in dogs with MMVD and CHF [12]. Similarly, analysis of plasma miRNA in Dachshunds with MMVD has shown a downregulation of known cardioprotective miRNAs (miR-30b, miR-133b) [13]. However, circulating miRNA likely originate not only from the mitral valve but are also contributed by the cardiac muscle or by other organs whose function is affected by poor cardiac output with cardiac dysfunction. In humans, Chen *et al*. have compared MMVD with fibroelastic deficiency and found decreases in miR-17, miR-1193, miR-1273e, miR-203, miR-28, miR-3065-5p, miR-4298, miR-505, miR-532, miR-646, miR-770, and miR-939 in myxomatous valves compared to fibroelastic deficient valves [14]. These studies showed that miRNA are likely involved in the development of MMVD but do not reconcile circulating miRNA with those of diseased tissues. Thus, there is a significant gap in knowledge concerning post-transcriptional dysregulation of gene expression in MMVD in humans and natural animal models. This has hampered the development of molecular targets in MMVD. The objectives of this study were to evaluate miRNA expression in VICs isolated from MMVD (diseased) versus non-MMVD (control) canine mitral valve tissues using next generation sequencing and RT-qPCR. We hypothesized that miRNA that underpin myxomatous pathology and the senescence processes would be highlighted in MMVD.

## Materials and methods

### Animals

The mitral valves were harvested from dogs donated to the Tufts University Cummings School of Veterinary Medicine Foster Hospital for Small Animals by their respective owners after humane euthanasia, and permission to use donated dog tissue was granted by Tufts University Cummings School of Veterinary Medicine Foster Hospital for Small Animals. The decisions for humane euthanasia made by the animal owners and their attending veterinarians were independent of this study. Reasons for euthanasia included chronic illnesses and behavior problems. Consent for the use of the donated bodies was acquired from all owners prior to tissue harvest following hospital policy. Dogs with history of neoplasia or were determined to have gross signs of neoplasia during necropsy and valve harvesting procedure were excluded from the study. In addition, dogs with congenital heart diseases or other acquired heart diseases other than MMVD were also excluded from the study. Mitral valves from a total of 20 dogs were harvested and used for this study.

### Valve characterization

Sections of the mitral valve were fixed in 4% paraformaldehyde. Valves were then embedded in paraffin, sectioned and stained with hematoxylin and eosin, Sirius red, Mason’s trichrome, and van Gieson’s stains. All sectioning were performed starting at the edge of the anterior leaflet. Based on the histologic morphology of the stained sections, the mitral valves were divided into three groups: normal, mildly diseased, and severely diseased. ‘Normal’ valves were defined as valve leaflets exhibiting no myxoid formation and maintaining the well-organized division of the three main layers within the mitral valve leaflet, namely the atrialis, spongiosa, and fibrosa layers. If any myxoid formation was seen within the spongiosa or fibrosa layers, the valve leaflet was classified as a diseased valve. Diseased valves were further categorized into mildly diseased or severely diseased groups based on the size of the myxomatous area per length of the valve leaflet. A section of at least a 1 cm leaflet length was examined for each sample, and myxomatous area were measured using Zen Blue imaging software [15]. The cumulative myxomatous area were determined for each valve sample. Leaflets with total myxomatous area less than 1 mm^2^/cm were classified as mild, and those with 1 mm^2^/cm or greater were classified as severe (S1 Fig).

Immunohistochemistry for alpha-smooth muscle actin (α-SMA) (Sigma-Aldrich Cat# A5228, RRID:AB_262054; monoclonal mouse antibody; lot # 091M4832; 5 μg/ml; 1:500 dilution; immunogen: N-terminal synthetic decapeptide of α-smooth muscle actin; reactivity against canine protein validated against mouse IgG_2a_ isotype, and Abcam Cat# ab5694; RRID:AB_2223021; polyclonal rabbit antibody; lot# GR131286-1; 0.2mg/ml; 1:100 dilution; immunogen: N-terminus of actin from human smooth muscle; reactivity against canine protein validated by manufacturer) and vimentin (Abcam Cat# ab8979, RRID:AB_306908; monoclonal mouse antibody; lot # GR264161-2; 1 μg/ml; 1:100 dilution; immunogen: cytoskeletal vimentin extract of calf lens; reactivity against canine protein validated by manufacturer) were performed on valve sections. Briefly, deparaffinized tissue sections were blocked in 3% BSA for 2 hours at room temperature followed by an incubation overnight using the respective primary antibodies or isotype at 4°C. The tissue sections were then incubated with fluorescent secondary antibody (Alexa Fluor) for 30 min at room temperature. DAPI (4′,6-diamidino-2-phenylindole) was applied to each section. A total of five images were analyzed, and only cells with focused DAPI staining and concurrent positive fluorescent signals on the acquired images were counted to ensure accurate identification of positively stained cells in the plane of focus.

### Valvular interstitial cell culture and isolation

Valvular interstitial cells were isolated from mitral valves using a digestion solution consisting of 7 U/ml collagenase I (Sigma-Aldrich), 7 U/ml collagenase XI (Sigma-Aldrich), 4 U/ml DNase (Sigma-Aldrich), and 4 U/ml hyaluronidase (Sigma-Aldrich). The digested cells were then filtered through a 70μm filter and cultured in αMEM media (Lonza) with 15% FBS (Hyclone), 0.3mg/ml L-glutamine (Corning), and 100U/ml penicillin and 100μg/ml streptomycin (Hyclone) onto untreated plastic culture plates. After 24 hours, the culture media is replaced to remove the unattached valvular endothelial cells. Cells were passaged once (P1) and cryopreserved for the subsequent analyses.

### Quantification of cell proliferation, colony formation, cell senescence, and response to TGFβ1/3 stimulation

Cell proliferation, colony-forming units (CFU), cell senescence, and response to transforming growth factor-β (TGF-β) 1 and 3 stimulations were compared among cell lines. The cell proliferation study was performed using the 3-(4,5-dimethylthiazol-2-yl)-2,5-diphenyltetrazolium bromide (MTT) assay (ATCC). Briefly, 5000 cells per well of passage 2 (P2) VICs were cultured in 48-well plates using αMEM with 10% FBS, 0.3mg/ml L-glutamine, and 100U/ml penicillin and 100μg/ml streptomycin. MTT assay absorbance was performed on days 1, 3, 5, and 7 after plating. Many of the cell lines were noted to be fully confluent between days 5 and 7. Therefore, MTT absorbance ratio between days 1 and 5 were compared and analyzed.

The CFU study was performed by plating 2000 P2 cells per 100 mm culture dish for 7 days in αMEM with 15% FBS, 0.3mg/ml L-glutamine, and 100U/ml penicillin and 100μg/ml streptomycin. Cells were then fixed with 2% glutaraldehyde and stained with 5% crystal violet. Colonies with more than 50 cells were counted as CFU.

The cell senescence study was performed with senescence-β-galactosidase (SA-β-gal) staining (Cell Signaling Technology). First, P2 VICs were cultured in 6-well plates using αMEM with 15% FBS, 0.3mg/ml L-glutamine, and 100U/ml penicillin and 100μg/ml streptomycin. Once the cells were approximately 80–90% confluent, cells were fixed and stained according to the manufacturer’s instructions. Five images were taken for each cell line, and the total number of cells per image and the number of positively stained cells were counted using ImageJ [16].

The TGF-β1 and TGF-β3 stimulation study was performed using P2 VICs cultured in αMEM with 15% FBS, 0.3mg/ml L-glutamine, and 100U/ml penicillin and 100μg/ml streptomycin. Once the cells were approximately 80% confluent, 20 ng/ml of either TGF-β1 (Cell Signaling Technology) or TGF-β3 (Cell Signaling Technology) was added to the media. After three days of TGF-β1 or TGF-β3 stimulation, the cells were harvested, and the mRNA was isolated using RNeasy kit (Qiagen) following the manufacturer’s instructions. cDNA was synthesized using miScript II RT kit (Qiagen) according to the manufacturer’s instructions. Expressions of α-SMA, vimentin, biglycan, decorin, lumican, collagen IA1 (Col1A1), elastin, connective tissue growth factor (CTGF), fibronectin, tenascin, p16, and p21 were analyzed using RT-qPCR. Sequences of the individual PCR primers are listed in Table 1. C_q_ numbers were first normalized using the average of two housekeeping genes (HPRT and RPS19). Fold change between stimulated and unstimulated expression changes were then calculated.

**Table 1:** mRNA primer sequences used in PCR analysis.

Primer	Forward Sequence	Reverse Sequence
α-SMA	tgcgtgacatcaaggagaag	tgctgttgtaggtggtctcg
Vimentin	ccgacaggatgttgacaatg	tcagagaggtcggcaaactt
Biglycan	cagaacaacgacatctcagagc	tcaccaggacgagagcgta
Decorin	cgctgtcagtgccatctc	gggggaagatcttttggtactt
Lumican	acctggaaattcttttaatgtatcatc	cggtatgtttttaagcttattgtagga
Col1A1	Qiagen canine primer (PPF00230A)	Qiagen canine primer (PPF00230A)
Elastin	tcactttctcttccggccac	gcctgggaattgggggtaaa
CTGF	tttaggaacagtgggagagc	catgaagaaggctggagaac
Fibronectin	aggttgttaccatgggca	gcataatgggaaaccgtgtag
Tenascin	ccacctcctacgacctgaga	ggatgcatttagccgtgcag
p21	Qiagen canine primer (PPF01045A)	Qiagen canine primer (PPF01045A)
P16	Qiagen canine primer (PPF11699A)	Qiagen canine primer (PPF11699A)
HPRT	agcttgctggtgaaaaggac	ttatagtcaagggcatatcc
RPS19	ccttcctcaaaaagtctggg	gttctcatcgtagggagcaag

### miRNA RT-qPCR and RNAseq

Total RNA, including mRNA and miRNA, was isolated from VICs (P2) cultured in αMEM with 15% FBS, 0.3mg/ml L-glutamine, and 100U/ml penicillin and 100μg/ml streptomycin using mirVana miRNA isolation kit (Invitrogen) following the manufacturer’s instructions. cDNA was synthesized using miScript II RT kit (Qiagen) according to the manufacturer’s instructions. RT-qPCR was performed using Canine miRNome miScript miRNA PCR Array (Qiagen), an array based on canine specific sequences in miRBase v.16, for the detection of 277 canine miRNAs. A total of 40 cycles of amplification were performed for RT-qPCR. C_q_ values within each sample were normalized using the geomean of all detected miRNA in that sample [17, 18]. Undetected miRNAs were assigned a C_q_ number of 40 prior to normalization.

Concurrently, miRNA sequencing (RNAseq) was performed. Total RNA was used as input for sequencing library preparation using TruSeq Small RNA Sample Preparation Kit (Illumina) per the manufacturer instruction. The resulted libraries were validate and quantified on a Fragment Analyzer (Advanced Analytical). The libraries were then sequenced on a HiSeq 2500 sequencer (Illumina) using High Output V4 chemistry and single read 50 bases format. The raw sequence results were processed into demultiplexed files in compressed fastq format using bcl2fq (Illumina). Adaptor sequences were trimmed from the resulted reads using Fastx Toolkits, and the trimmed reads were analyzed using miPRO2.

### Statistical and data analysis

Statistical analysis was performed using commercially available software (SPSS) [19]. Kruskal-Wallis test was used for non-parametric statistical testing of differences among more than two groups, and Mann-Whitney U test or t-test (normally distributed data) was used for two-way comparisons. Targets were predicted *in silico* using a combination of TargetScan [20] and miRDB [21]. Hierarchical clustering (HCL) and principal component analysis (PCA) were performed using Multiexperimental Viewer (MeV 4.9.0) [22].

## Results

### Demographic profile and MMVD classification of subjects

**Fig 1:**
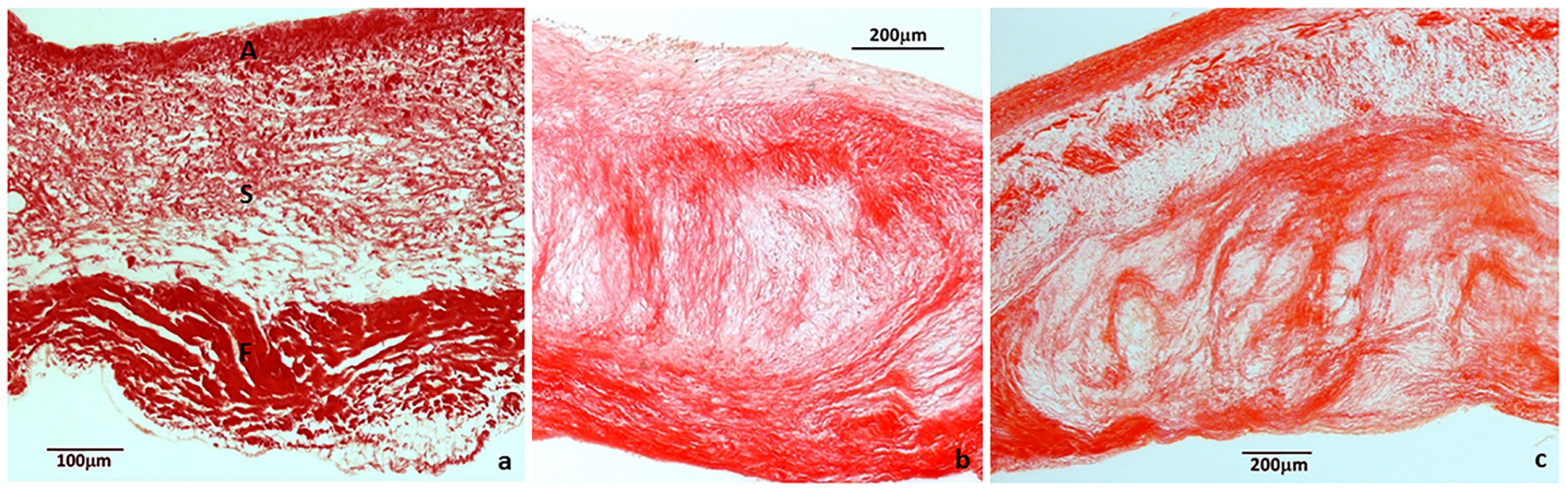
Examples images of valve leaflet sections. Histologic sections of a) normal valve, b) mildly diseased valve, and c) severely diseased valve at the edge of the mitral valve leaflet. The three distinct layers of a normal valve can be seen in the normal valve leaflet, including the atrialis (A), spongiosa (S), and fibrosa (F) layers. The division of these layers was interrupted by nodular formation in the mildly and severely diseased valves, with larger myxomatous area observed in the severely diseased valves.

Mitral valves from 20 dogs were harvested for this study. The demographic profiles are shown in Table 2. The collected mitral valves were divided into three classification groups based on histologic changes: Normal valves (n = 5), mildly diseased (n = 7), and severely diseased (n = 8) (Fig 1).

**Table 2:** Demographic profile of study dogs.

Disease Status	Total	Median Age (Range) Years	Breed	Total
Normal	5	4	Beagle	2
		(2–10)	German Shepherd	2
			Doberman Pincher	1
Mildly Diseased	7	7	Mastiff	1
		(3–10)	Chihuahua	1
			Maltese	1
			Mixed Breed	2
			Yorkshire Terrier	1
			Corgi	1
Severely Diseased	8	12.5	German Shepherd	1
		(7–17)	Chihuahua	1
			Mixed Breed	5
			English Bulldog	1

The dogs in the severely diseased group were significantly older than those in the normal group (p = 0.009) and the mildly diseased group (p = 0.012). There is no significant difference in age between the mildly diseased and normal groups.

### Valve and valvular interstitial cell characterization

#### Diseased valves have higher expression of α-SMA and lower expression of vimentin

**Fig 2:**
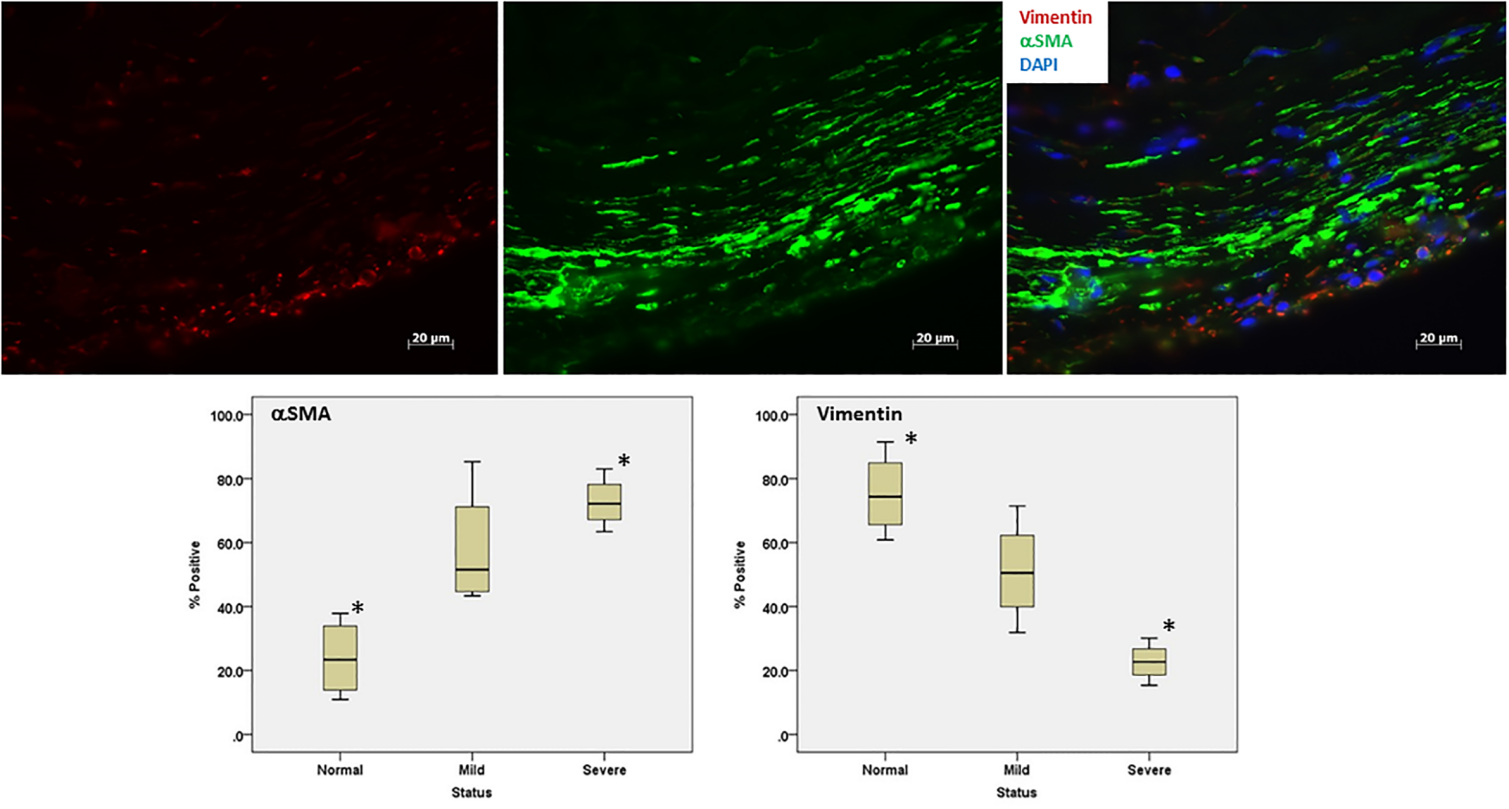
Immunohistochemistry staining for α-SMA and vimentin. The top panels show representations of the expression level of vimentin and α-SMA in a severely diseased valve. Diseased valves have cells with increased α-SMA expression and decreased vimentin expression (vimentin: rhodamine/red, α-SMA: FITC/green, DAPI/blue). The lower panels show the difference in the number of cells positive for α-SMA (left) and vimentin (right) from valves sections of normal, mildly, and severely diseased valves. Asterisks denote changes that are statistically significant.

Based on immunohistochemistry, the severely diseased valves have a significantly higher number of α-SMA positive cells (p = 0.018) and a fewer number of vimentin positive cells (p = 0.01) than normal valves (Fig 2). However, there were no significant differences between normal and mildly diseased valves or between mildly or severely diseased valves.

#### Diseased VICs have decreased cell proliferation compared to VICs from normal valves

**Fig 3:**
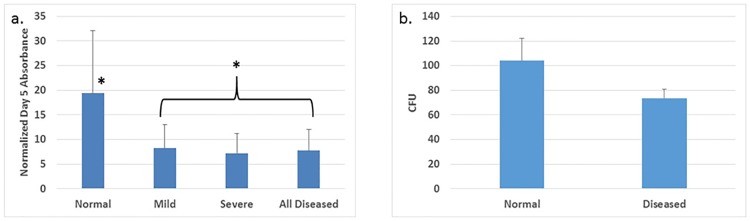
Cell proliferation and CFU. a) Day 5 MTT absorbance normalized by Day 1 absorbance after cell plating for VICs from normal, mildly, and severely diseased valves, and a combination of all of the diseased VICs. Asterisks denote changes that were statistically significant. b) CFU from VICs harvested from normal and diseased valves.

Valvular interstitial cells isolated from diseased valves had decreased cell proliferation compared to those from normal valves. We compared the ratio of day 5 to day 1 absorbance using the MTT assay. The increases in absorbance, an indication of cell proliferation and cell viability, from day 5 compared to day 1 were significantly lower for VICs from both mildly (n = 8) or severely (n = 7) diseased valves compared with VICs from normal (n = 5) valves (Fig 3a) (p = 0.005). There was no statistically significant decrease in the diseased VIC’s ability to form CFU (p = 0.054) (Fig 3b).

#### Diseased VICs have increased expression of senescence markers

**Fig 4:**
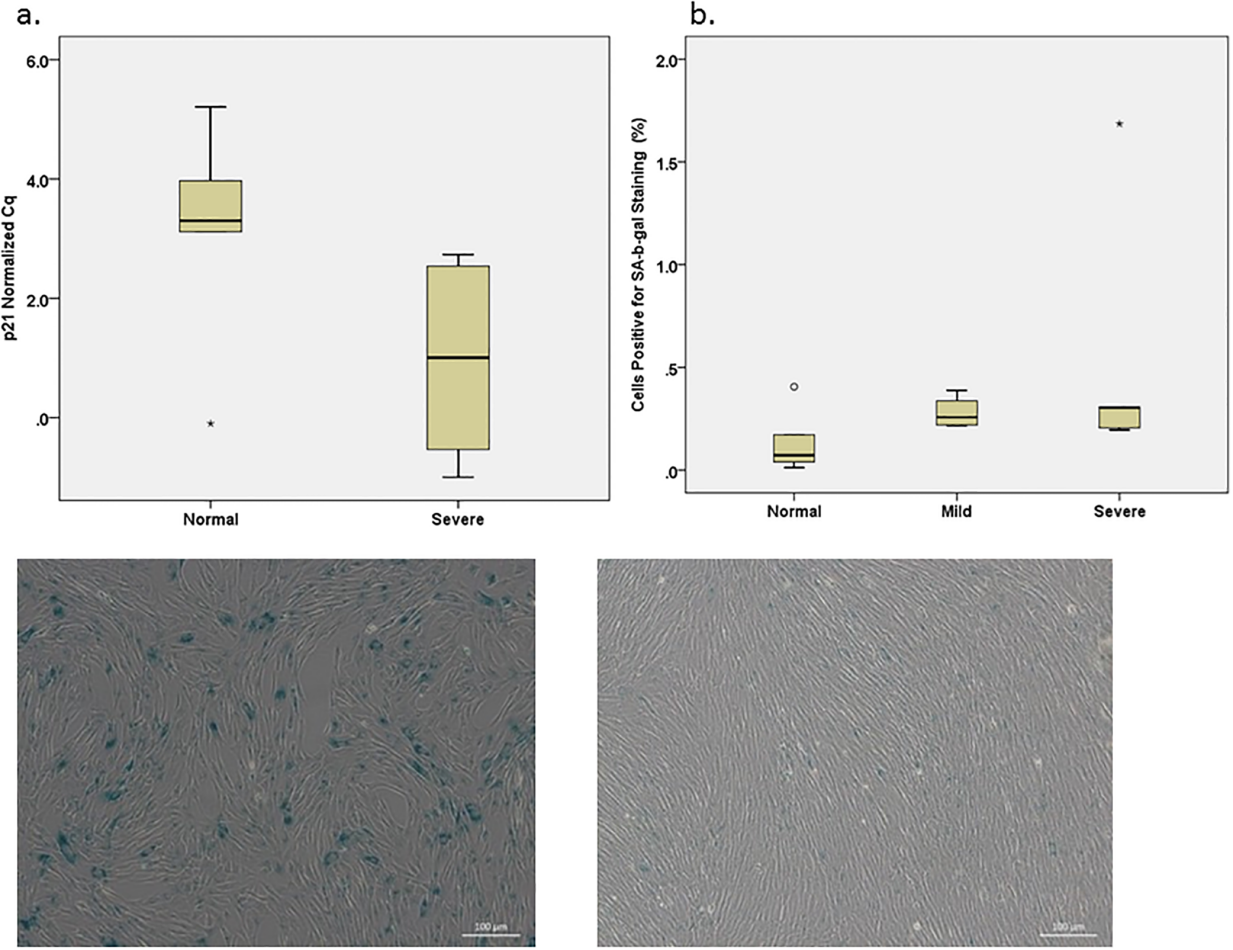
Senescence marker in VICs. a) Box plot illustrating differences in RT-qPCR C_q_ number for p21 between VICs harvested from normal and severely diseased valves. (*) denote outliers. b) Box plot illustrating the percentage of cells positive for SA-β-gal staining from normal, mildly, and severely diseased valves. (*) and (o) denote outliers.

Valvular interstitial cells from severely diseased valves, (n = 8), had increased expression of p21 compared with those from normal valves (n = 5) (p = 0.04) based on RT-qPCR (Fig 4a). There was no statistical difference in p16 expression between disease categories. In addition, there were no statistically significant differences of the amount of SA-β-gal stain uptake for VICs from normal, mildly, and severely diseased valves (Fig 4b).

#### VICs differentiation to myofibroblastic phenotype induced by exposure to TGF-β1 or TGF-β3 stimulation

**Fig 5:**
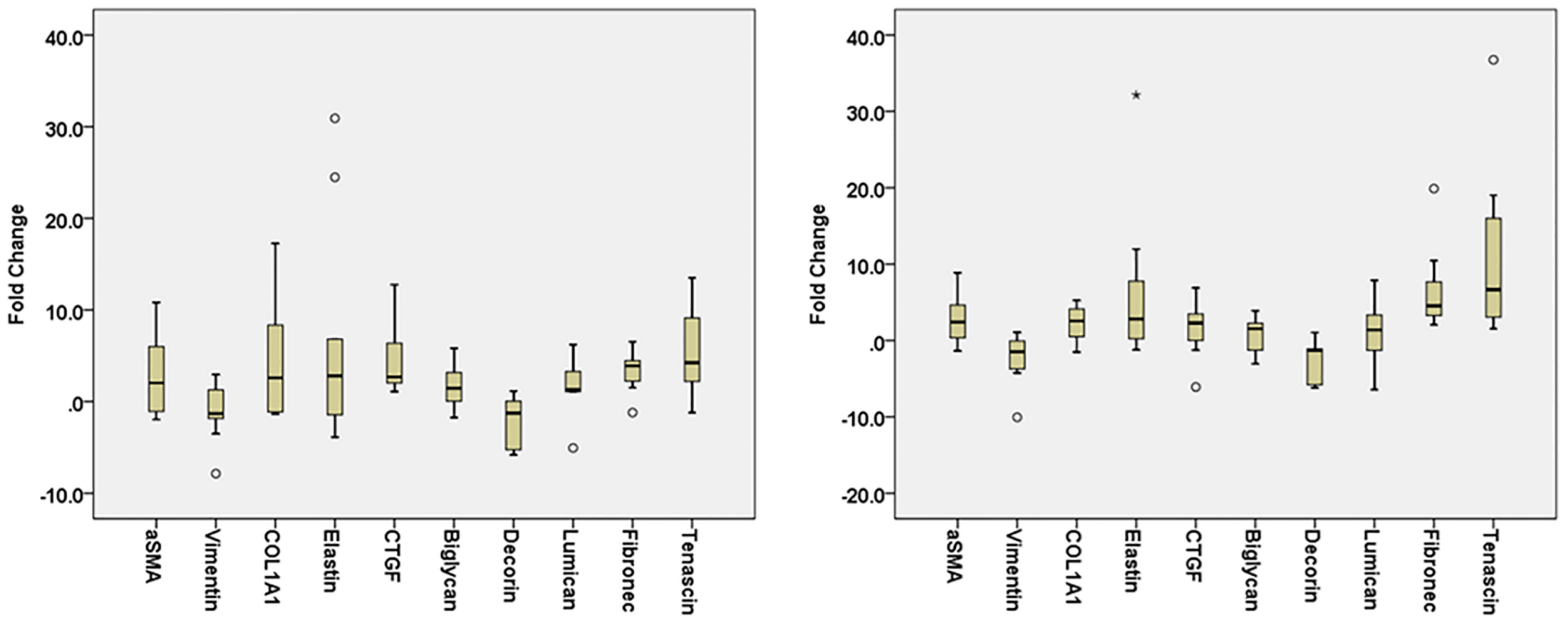
mRNA expression fold change after TGF-β1/3 stimulation. Box plot illustrating the expression fold change based on RT-qPCR for VICs after stimulation with either a) TGF-β1 or b) TGF-β3. (o) and (*) denote outliers.

Stimulation of VICs from both normal and diseased valves (n = 20) with TGF-β1/3 resulted in increases in the median mRNA expression fold change (fold change > 0) of α-SMA, ColIA1, elastin, CTGF, biglycan, fibronectin and tenascin, and decreases in the median fold change (fold change < 0) of vimentin and decorin (Fig 5). These data indicate that canine VIC employed in these experiments have potential for induction of myofibroblastic differentiation. There were no statistical difference in the expression change in any of the tested mRNA between disease groups.

### miRNA expression profile varies significantly between VICs from normal, mildly diseased, and severely diseased valves

#### miRNAs were differentially expressed between normal and diseased valves using RT-qPCR analysis

The average number of miRNAs detected for each cell line was 77% of the 277 miRNAs tested using Canine miRNome miScript miRNA PCR Array. Twenty-four of the 277 (8.7%) miRNAs included in the RT-qPCR analysis were differentially expressed among the three groups (Kruskal-Wallis, p<0.05). These miRNAs are listed in Table 3. When comparing the severely diseased group with the two other groups (normal and mildly diseased), 28 (10.1%) miRNAs were differentially expressed (Mann-Whitney U, p<0.05), and when comparing the mildly diseased group and the severely diseased group, 46 (16.6%) miRNAs were differentially expressed (Mann-Whitney U, p<0.05).

**Table 3:** List of miRNAs that were differentially expressed between the disease groups based on RT-qPCR.

Disease Group Compared	Differentially expressed miRNA
Normal vs. mild vs. severe (p<0.05)	cfa-let-7a, cfa-let-7b, cfa-let-7c, cfa-let-7f, cfa-miR-127, cfa-miR-1271, cfa-miR-130a, cfa-miR-139, cfa-miR-17, cfa-miR-1836, cfa-miR-1837, cfa-miR-20a, cfa-miR-23a, cfa-miR-25, cfa-miR-26a, cfa-miR-29b, cfa-miR-378, cfa-miR-421, cfa-miR-502, cfa-miR-503, cfa-miR-542, cfa- miR-652, cfa-miR-653, cfa-miR-872
Normal and mild vs. severe (p<0.05)	cfa-let-7a, cfa-let-7b, cfa-let-7c, cfa-let-7f, cfa-miR-103, cfa-miR-127, cfa-miR-130a, cfa-miR-130b, cfa-miR-134, cfa-miR-139, cfa-miR-152, cfa-miR-17, cfa-miR-190b, cfa-miR-191, cfa-miR-195, cfa-miR-20a, cfa-miR-21, cfa-miR-222, cfa-miR-23a, cfa-miR-25, cfa-miR-26a, cfa-miR-32, cfa-miR-361, cfa-miR-378, cfa-miR-421, cfa-miR-542, cfa-miR-761, cfa-miR-9
Mild vs. severe (p<0.05)	cfa-let-7a, cfa-let-7b, cfa-let-7c, cfa-let-7f, cfa-let-7g, cfa-miR-103, cfa-miR-1271, cfa-miR-128, cfa-miR-130a, cfa-miR-130b, cfa-miR-134, cfa-miR-135a, cfa-miR-139, cfa-miR-15b, cfa-miR-17, cfa-miR-181a, cfa-miR-181b, cfa-miR-181d, cfa-miR-1835, cfa-miR-1837, cfa-miR-191, cfa- miR-195, cfa-miR-20a, cfa-miR-21, cfa-miR-222, cfa-miR-23a, cfa-miR-25, cfa-miR-26a, cfa-miR-26b, cfa-miR-30d, cfa-miR-328, cfa-miR-34b, cfa-miR-363, cfa-miR-378, cfa- miR-421, cfa- miR-423a, cfa-miR-450b, cfa-miR-454, cfa-miR-494, cfa-miR-502, cfa-miR-532, cfa-miR-543, cfa-miR-578, cfa-miR-652, cfa-miR-653, cfa-miR-872

#### miRNAs were differentially expressed between normal and diseased valves using RNAseq analysis

The average number of miRNAs detected by sequencing for each dog was 55% of the 453 miRNAs (miRBase, v. 21), with counts fewer than 10 reads treated as “no detection.” Thirty-six of the 453 (7.9%) miRNAs analyzed in the RNAseq analysis were significantly differentially expressed for the three groups (Kruskal-Wallis, p<0.05). These miRNAs are listed in Table 4. When comparing the severely diseased group with the two other groups (normal and mildly diseased), 15 (3.3%) miRNAs were differentially expressed (Mann-Whitney U, p<0.05), and when comparing the mildly diseased group and the severely diseased group, 21 (4.6%) miRNAs were differentially expressed (Mann-Whitney U, p<0.05).

**Table 4:** List of miRNAs that were differentially expressed between the disease groups based on RNA sequencing.

Disease Group Compared	Differentially expressed miRNA
Normal vs. mild vs. severe (p<0.05)	cfa-let-7d, cfa-miR-101, cfa-miR-106b, cfa-miR-10a, cfa-miR-1296, cfa-miR-1306, cfa-miR-1307, cfa-miR-132, cfa-miR-1343, cfa-miR-136, cfa-miR-153, cfa-miR-17, cfa-miR-181a, cfa-miR-1841, cfa-miR-18a, cfa-miR-190b, cfa-miR-192, cfa-miR-197, cfa-miR-19a, cfa-miR-19b, cfa-miR-203, cfa-miR-20a, cfa-miR-22, cfa-miR-224, cfa-miR-2387, cfa-miR-27a, cfa-miR-30d, cfa-miR-328, cfa-miR-34c, cfa-miR-497, cfa-miR-499, cfa-miR-500, cfa-miR-574, cfa-miR-8903, cfa-miR-93, cfa-miR-99b
Normal and mild vs. severe (p<0.05)	cfa-let-7c, cfa-miR-10a, cfa-miR-1307, cfa-miR-132, cfa-miR-136, cfa-miR-181a, cfa-miR-181b, cfa-miR-196b, cfa-miR-20a, cfa-miR-30d, cfa-miR-33b, cfa-miR-34c, cfa-miR-497, cfa-miR-499, cfa-miR-676
Mild vs. severe (p<0.05)	cfa-let-7d, cfa-miR-101, cfa-miR-10a, cfa-miR-1296, cfa-miR-1306, cfa-miR-1307, cfa-miR-130a, cfa-miR-136, cfa-miR-17, cfa-miR-181b, cfa-miR-196b, cfa-miR-197, cfa-miR-215, cfa-miR-22, cfa-miR-30d, cfa-miR-33b, cfa-miR-497, cfa-miR-503, cfa-miR-574, cfa-miR-628, cfa-miR-676

#### Changes in miRNA expression levels differed between RT-qPCR and RNAseq

**Fig 6:**
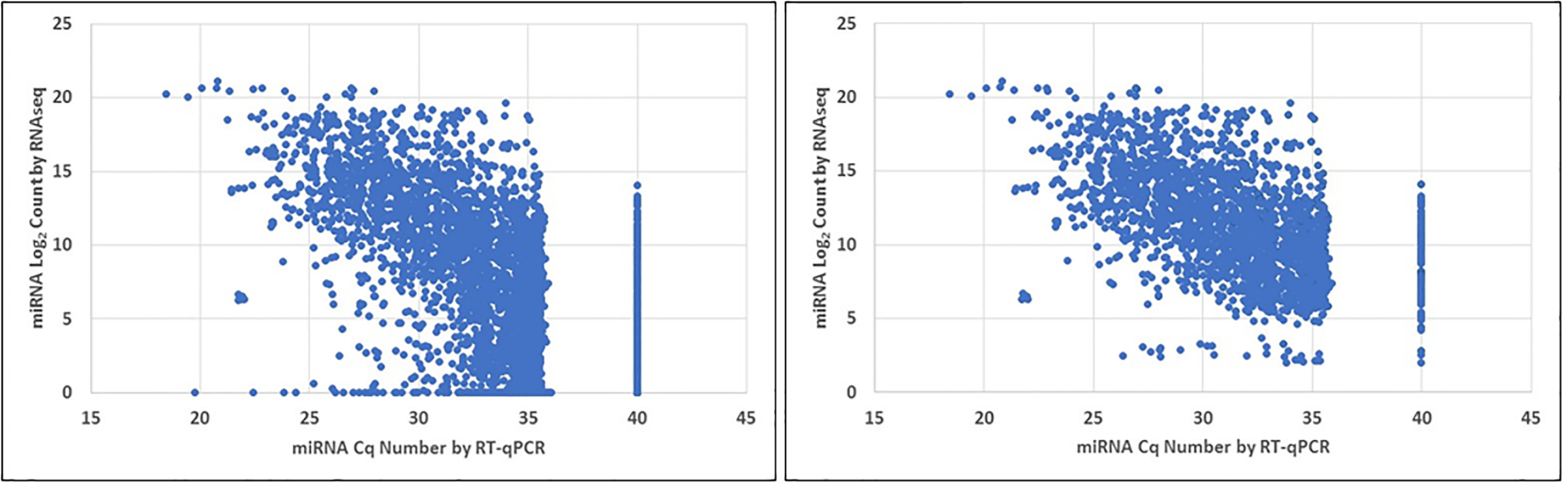
Relation between normalized RNAseq count and normalized RT-qPCR C_q_ number for individual miRNAs. High RNAseq log_2_ count and low C_q_ number represent highly expressed miRNA. Increased divergence between the two methods was seen for miRNA with lower expression levels (left panel). The right panel illustrates improvement in divergence if miRNA with low count number (RNAseq count number <50) are removed from analysis. The data points corresponding to a C_q_ number of 40 represented miRNAs that were not detected by RT-qPCR.

Comparing the miRNA differential expression analyses between disease states obtained by RT-qPCR and RNAseq, we observed discordances between the two methods. As illustrated in Fig 6, the divergence between level of expression and count number was more evident for those miRNA with lower expression levels (either low RNAseq log_2_ count or high C_q_ number), suggesting that accuracy may be confounded by either method with low miRNA copy numbers. We therefore did not further analyze any miRNA with RNAseq read number less than 50 counts.

#### Let-7c, miR-17, miR-20a, and miR-30d were decreased in VICs of diseased valves

**Fig 7:**
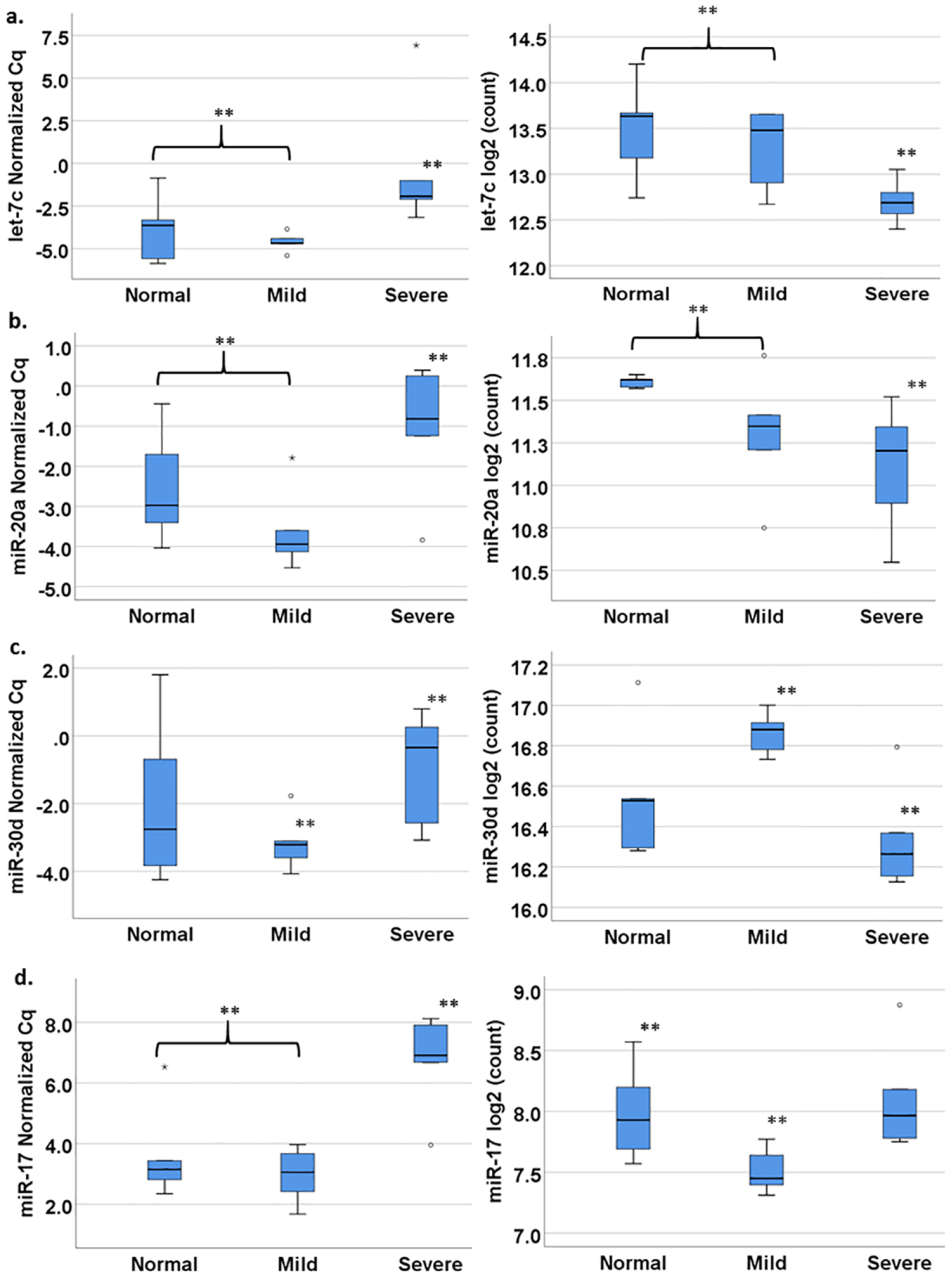
Normalized RT-qPCR C_q_ number and RNAseq count number for let-7c, miR-17, miR-20a, and miR-30d. Both let-7c (a) and miR-20a (b) expressions were lower in VICs from severely diseased valves compared with the cells from normal or mildly diseased valves. A decrease in miR-30d expression was seen when comparing between VICs of mildly diseased and severely diseased valves (c). For miR-17, RT-qPCR showed a decrease in expression in VICs from severely diseased valves compared with both that of normal and mildly diseased valves, but significant decrease was only seen in RNAseq when comparing between mildly diseased and normal valves (d). High C_q_ number or low log_2_ count represent low expression levels. (°) and (*) denote outliers. (**) indicate statistical significance.

Based on the results from both RNAseq and RT-qPCR, four miRNAs were differentially expressed (p<0.05) in VICs of diseased versus normal valves using both analysis methods. All four miRNAs were found in moderate to high copy numbers in VICs. When comparing VICs from severely diseased valves with those from normal and mildly diseased valves, decreases in let-7c (p = 0.011 PCR, p = 0.019 RNAseq, PCR fold change (FC) = -15.7) (Fig 7a) and miR-20a (p = 0.041 PCR, p = 0.040 RNAseq, FC = -4.0) (Fig 7b) expression levels were noted. When comparing between VICs from severely diseased valves and mildly diseased valves, a decrease in miR-30d expression level was seen (p = 0.031 PCR, p = 0.031 RNAseq, FC = -3.8) (Fig 7c). miR-17 levels were decreased in VICs from severely diseased valves compared with either VICs from normal or mildly diseased valves based on RT-qPCR analysis (p = 0.005, FC = -7.4), but this decrease was only significant when comparing between mildly diseased and normal valves based on RNAseq analysis (p = 0.048) (Fig 7d). Given the increase in median age for the dogs in the mildly and severely diseased group, we have also analyzed the effect of age on miRNA expression levels and did not find any age-associated change in let-7c, miR-17, miR-20a, and miR-30d.

**Fig 8:**
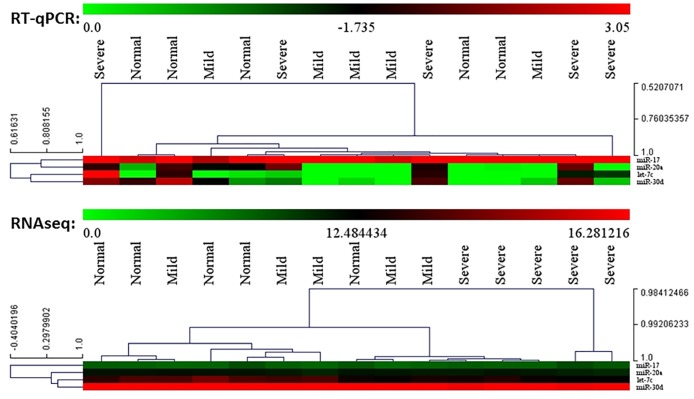
Hierarchical clustering of let-7c, miR-17, miR-20a, and miR-30d using both RT-qPCR and RNAseq data. Close clustering of VICs from severely diseased valves was clearly seen when analyzing the expression level based on RNAseq data. However, this clustering was not appreciated when analyzing the RT-qPCR data, with greater inter-node distance between VICs from severely diseased valves. For the RT-qPCR scale, red denotes low expression and green denotes high expression. For the RNAseq scale, red denotes high counts and green denotes low counts.

**Fig 9:**
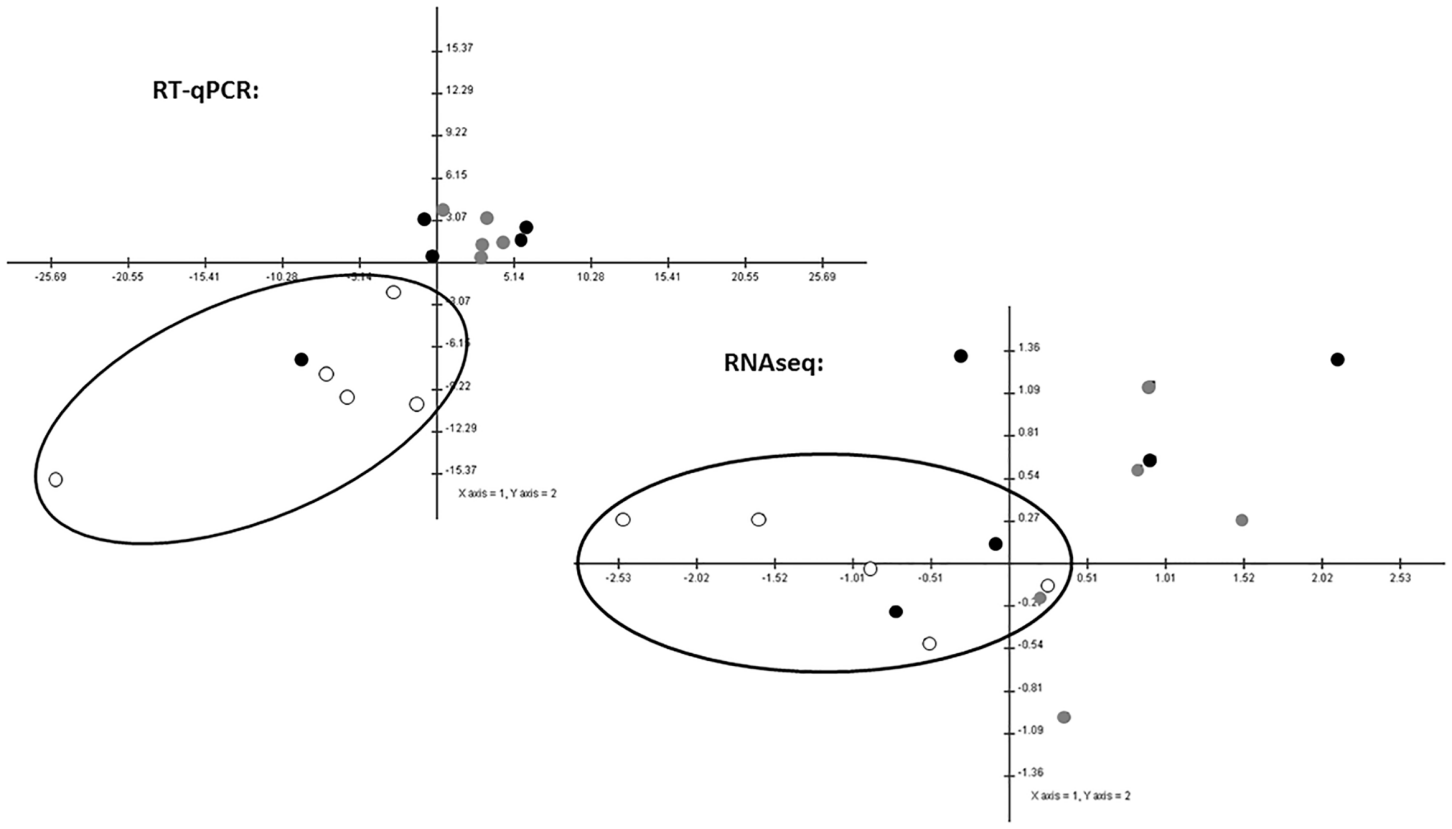
Principal component analysis of let-7c, miR-17, miR-20a, and miR-30d using both RT-qPCR and RNAseq data. Samples from severely diseased valves appeared to be closely clustered when analyzing the RT-qPCR data. Analysis of the RNAseq data showed greater dispersion of samples from severely diseased valves. Axis 1 (x-axis) and axis 2 (y-axis) are shown. Solid black circles represent samples from normal valves, solid gray circles represent samples from mildly diseased valves, and open circles represent samples from severely diseased valves.

PCA and HCL analyses of the four miRNAs of interest (let-7c, miR-17, miR-20a, and miR-30d) using both RT-qPCR and RNAseq data also supported the observed miRNA differences between VICs from severely diseased valves and those from normal and mildly diseased valves. HCL plot based on RNAseq data showed clear separation of VICs from severely diseased valves from the other two groups, although this separation was not as clear based on the RT-qPCR data (Fig 8). However, PCA using RT-qPCR data showed closer association and clustering of severely diseased valves while greater separation between severely diseased valves was seen when the analysis was performed using the RNAseq data (Fig 9). Taken together, The PCA and HCL analyses showed similarity and low variance for the four miRNA expression within disease categories.

## Discussion

In this study of canine MMVD, we observed dysregulated miRNAs associated with the control of myofibroblastic differentiation, extracellular matrix (let-7c, miR-17, miR-20a), and senescence and apoptosis (miR-17, miR-20a, miR-30d) [23–31]. These data are consistent with the observed valvular tissue architecture, phenotype, and age-related senescence of VIC, and provides insight into potential mechanisms by which MMVD progresses with age.

Consistent with the observation that MMVD is an age-related disease [2], dogs affected by MMVD in our study were older than those in the other two groups. In addition, VICs from diseased valves show characteristics of myofibroblasts including an increase in α-SMA and decrease in vimentin expressions. We also observed a decrease in cell proliferation and viability, and an increase in the expression of p21, a cyclin-dependent kinase inhibitor that may trigger cell cycle arrest or lead to cell senescence or apoptosis [32]. The VIC from healthy dog valves have the capacity to differentiate to myofibroblastic cells, which was evidenced by their response to TGF-β1/3, evoking increased α-SMA expression and increased production of extracellular matrix components including collagen, elastin, CTGF, fibronectin, and tenascin. This type of response to TGF-β has similarly been observed in human VIC [33]. These extracellular matrix changes and differentiation into myofibroblastic cells are central to the development of diseased canine mitral valves with disruption of the organized structure of the valve leaflets. In addition, miRNA profiling of VICs using both RT-qPCR and RNAseq showed decreases in expressions of let-7c, miR-17, miR-20a, and miR-30d.

### Decreases in miR-20a and miR-17 are associated with cell senescence

Based on the RT-qPCR and RNAseq analyses, decreases in miR-20a and miR-17 expressions were noted in VICs harvested from severely affected valves. The decrease in miR-17 expression seen in MMVD valves was similar to the findings by Chen *et al*., although in that study, the MMVD valves were compared to valves affected by fibroelastic deficiency [14]. The decrease in miR-20a was noted consistently in both the RT-qPCR and RNAseq analyses. On the other hand, the RT-qPCR data show a more robust decrease in miR-17 with disease development than the RNAseq data, where a statistical significant decrease was noted only in cells from mildly diseased valves.

Both miR-20a and miR-17 belong to the miR-17-92 cluster, where miR-20a and miR-17 can be grouped together as one functional unit [34]. This is a well-studied gene cluster that has been implicated in regulation of cell senescence and apoptosis, endothelial-to-mesenchymal transition (EndMT), epithelial-to-mesenchymal transition, and fibrosis [24–26, 28, 30, 31, 34, 35]. Frank *et al*. showed that miR-20a can control apoptosis of cardiomyocytes through the inhibition of EGLN3 and E2F1 [34]. In the canine species, EGLN3 and E2F1 are predicted targets of miR-20a [20, 21]. Similarly, miR-20a expression was found to be downregulated in senescing human diploid fibroblasts, and it may exert its anti-senescence effects through the p21 and p16 pathways [28]. In fact, the entire miR-17-92 cluster is commonly found to be downregulated in senescent cells, and decreases in both miR-20a and miR-17 have been correlated with the increase in transcription of p21 [26]. p21 is a potent, tight-binding inhibitor of CDKs that is needed to proceed from the G1 checkpoint into the S phase for cell cycling [27]. As p21 increases, G1 checkpoint and cell cycle arrest are activated [30].

The critical role that miR-20a and miR-17 play in controlling cell senescence is of interest in the canine MMVD model. Not only did we show that these two miRNA were down-regulated with disease, we also observed increased cell senescence markers (increase in p21 expression) and decreased cell viability and growth. In addition, this same decrease in miR-17 was found in human myxomatous mitral valves when comparing to valves affected by fibroelastic deficiency [14]. To directly link miR-20a and miR-17 to these observations, a gain and loss of function test will need to be performed.

### Downregulation of miR-20a, miR-17, and let-7c is associated with TGF-β signaling

TGF-βR1/2 are direct targets of miR-20a and miR-17 [24, 35], and for the canine species, TGF-βR is also a predicted target for these two miRNA [20, 21]. Previous studies have also showed a decrease in miR-20a during EndMT [35].

With regards to the observed let-7c level in VICs, RT-qPCR and RNAseq analyses showed that this miRNA is downregulated in cells from severely diseased valves when comparing with normal and mildly diseased valves. Similar to miR-20a and miR-17, let-7c has also been found to target TGF-βR1 [23], and it was reported to be a key inhibitor for fibroblast proliferation and migration during wound healing [36].

Given that TGF-β had been implicated in MMVD in people [9], the interaction of miR-20a, miR-17, and let-7c with TGF-βR is of particular interest to myxomatous mitral valve development. The valves showed characteristics of myofibroblastic differentiation with increased α-SMA and decreased vimentin expressions. In addition, VICs were responsive to TGF-β1/3 stimulation, exhibiting changes typical of myofibroblasts. The ability of miR-20a, miR-17, and let-7c to control the phenotypic transformation of VICs into myofibroblasts will need to be tested with overexpression of these three miRNAs in VICs.

### Loss of miR-30d and putative dysregulation of apoptosis

RT-qPCR and RNAseq analyses showed a decrease in miR-30d expression in VICs from severely diseased valves compared with mildly diseased valves. Melman *et al*. showed that miR-30d may play a role in protecting cells from apoptosis by targeting mitogen-associated kinase 4 (MAP4K4), a downstream effector of tumor necrosis factor (TNF). And in their canine model, they showed a decrease in miR-30d expression in coronary blood was seen tachypacing-induced heart failure [29]. It may therefore help mitigate responses to TNF-mediated inflammatory response that warrants further investigation.

### Discordance between RT-qPCR and RNAseq analyses

Let-7c, miR-17, miR20a, and miR-30d were the four miRNAs out of the 275 analyzed by both RT-qPCR and RNAseq that showed differential expression (p<0.05) between normal and diseased cells with the same direction in change (up- or down-regulated) by both methods. Discordance between RT-qPCR and RNAseq, in terms of difference in differential expression analysis and inconsistent fold change directions, have been previously reported [37, 38]. Mestdagh *et al*. have systematically analyzed different miRNA expression platforms. They have found that agreement on the number of differentially expressed miRNA between two platforms of different technology (i.e. qPCR and sequencing) varied between 35–63% for the 12 platforms that were tested [39]. This discordance is the net result of varying sensitivity and specificity characteristics inherent to each platform. For RT-qPCR, varying degrees of cross-hybridization of miRNA family members or reduced discrimination between unprocessed and mature forms of the miRNAs may occur [37]. RNAseq is more accurate at differentiating small variations in sequences, which are commonly seen between members of the same family of miRNA [37]. On the other hand, sequencing errors can occur, and the need for adaptor removal and library preparation may bias the results [37]. Differences between the two analysis results were notably more frequent for miRNAs of low abundance as illustrated in Fig 6 by the increase in spread in correlation between the RNAseq log_2_ count number and RT-qPCR C_q_ number. Because our candidate miRNA (let-7c, miR-17, miR-20a, and miR-30d) were deemed differentially expressed by both techniques, we believe that the probability for false positive or spurious changes is decreased, thus strengthening our findings.

One potential limitation with our study is the lack of breed and age matching between the normal and diseased groups. Given that the classification of the mitral valves was performed histologically, the differences in miRNA expression as a function of histologic changes remain valid. It is also not surprising to see an increase in age for the diseased group of dogs since canine MMVD is an age-related disease with close to 100% of older dogs, regardless of breed, exhibiting myxomatous valvular changes. Nevertheless, the expression level of the four miRNA of interest were shown to be independent of the age group.

Using both RT-qPCR and RNAseq to analyze miRNA differential expression may also increase the number of false negative findings and potentially overlook some miRNA that may be involved in MMVD development. For example, any technical limitation in detection by one technique will likely eliminate the candidate miRNA. However, this more stringent selection approach may minimize the number of false positive miRNA.

The four miRNAs noted to change in VICs with disease development were not seen to vary in plasma exosomal miRNA based on our previous work. Given that circulating plasma exosomes are contributed by all organs, it is not surprising that changes originated from the mitral valve, a small part of the heart, may not be easily detected within the plasma. In addition, it is unclear if let-7a, miR-17, miR-20a, and miR-30d are differentially sorted into exosomes by VICs.

Lastly, the sample size of this study is small and this represents a pilot study, further verification is necessary in a larger replicative study.

## Conclusions

We have demonstrated that miRNA dysregulation (let-7c, miR-17, miR-20a, and miR-30d) may participate in canine MMVD development, and these miRNA should be explored further as potential therapeutic targets. In future studies, whole valvular tissue from dogs naturally affected with MMVD could be explored given that the *in vitro* setting of this study may influence the molecular characteristics of the VICs. Specifically, loss and gain of function testing with these four miRNAs using *in vitro* studies will be critical to rigorously verify their biological function and role in cell senescence.

## Supporting information

S1 Fig Method in determining valve myxomatous area. The same histology sections shown in Fig 1 in addition to illustrating how myxomatous areas were determined. The dark lines are drawn to encircle a myxomatous area.
(TIF)

S1 Table Comparison of grading based on histopathology and Whitney classification. (DOCX)
