# Supplementary figures and images for: Dysregulation of valvular interstitial cell let-7c, miR-17, miR-20a, and miR-30d in naturally occurring canine myxomatous mitral valve disease
Source: PLoS One. 2018 Jan 9;13(1):e0188617. doi: 10.1371/journal.pone.0188617 (PMC5760013; doi:10.1371/journal.pone.0188617)

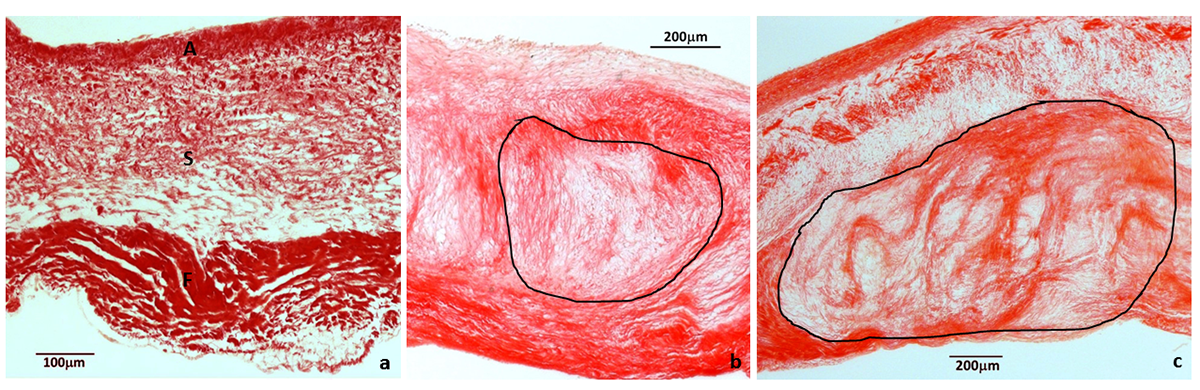

Supplement: S1 Fig — The same histology sections shown in Fig 1 in addition to illustrating how myxomatous areas were determined. The dark lines are drawn to encircle a myxomatous area. (TIF) [file pone.0188617.s001.tif]
