# Supplementary material for: Dysregulation of valvular interstitial cell let-7c, miR-17, miR-20a, and miR-30d in naturally occurring canine myxomatous mitral valve disease
Source: PLoS One. 2018 Jan 9;13(1):e0188617. doi: 10.1371/journal.pone.0188617 (PMC5760013; doi:10.1371/journal.pone.0188617)
Supplement: S1 Table — (DOCX) [file pone.0188617.s002.docx]

**Table S1. Comparison of grading based on histopathology and Whitney classification**

| **Grading Based on Histopathology** | **Grading Based on Whitney Classification** |
| --- | --- |
| Normal (n=5) | 0 (n=5) |
| Mild (n=5) | 1 (n=1), 2 (n=4) |
| Severe (n=5) | 3 (n=2), 4 (n=3) |
